# Computer Simulation and Field Experiment for Downlink Multiuser MIMO in Mobile WiMAX System

**DOI:** 10.1155/2015/481676

**Published:** 2015-09-02

**Authors:** Kazuhiro Yamaguchi, Takaharu Nagahashi, Takuya Akiyama, Hideaki Matsue, Kunio Uekado, Takakazu Namera, Hiroshi Fukui, Satoshi Nanamatsu

**Affiliations:** ^1^Department of Computer and Media Engineering, Tokyo University of Science, Suwa 5000-1, Toyohira, Chino-shi, Nagano 391-0292, Japan; ^2^Azumino City, Nagano, Japan; ^3^MIRAIT Technologies Corporation, Japan; ^4^NPO CCC21L, Japan

## Abstract

The transmission performance for a downlink mobile WiMAX system with multiuser multiple-input multiple-output (MU-MIMO) systems in a computer simulation and field experiment is described. In computer simulation, a MU-MIMO transmission system can be realized by using the block diagonalization (BD) algorithm, and each user can receive signals without any signal interference from other users. The bit error rate (BER) performance and channel capacity in accordance with modulation schemes and the number of streams were simulated in a spatially correlated multipath fading environment. Furthermore, we propose a method for evaluating the transmission performance for this downlink mobile WiMAX system in this environment by using the computer simulation. In the field experiment, the received power and downlink throughput in the UDP layer were measured on an experimental mobile WiMAX system developed in Azumino City in Japan. In comparison with the simulated and experimented results, the measured maximum throughput performance in the downlink had almost the same performance as the simulated throughput. It was confirmed that the experimental mobile WiMAX system for MU-MIMO transmission successfully increased the total channel capacity of the system.

## 1. Introduction

Recently, increasing channel capacity and improving system performance are serious challenges for high speed wireless communication systems. To overcome these, multiple-input multiple-output (MIMO) techniques [1, 2] are employed such as long term evolution (LTE), IEEE 802.11n, and Worldwide Interoperability for Microwave Access (WiMAX). Multiple antennas can be used for increasing data rates through multiplexing or for improving performance through diversity such as in single user MIMO (SU-MIMO) systems. Multiuser systems with multiple antennas at the transmitters and/or receivers are called multiuser MIMO (MU-MIMO) systems. The SU-MIMO system can improve the channel capacity for only one user; however, there is some limitation in increasing the number of receiving antennas on mobile stations. The MU-MIMO [3] system can also improve the total channel capacity by increasing the number of mobile stations, and MU-MIMO systems have been already researched for wireless communications with high wireless link capacity such as LTE-Advanced and IEEE 802.11ac technologies.

Many researchers have reported field experimental results with the 3.5-GHz frequency band [4, 5]. The 2.5-GHz frequency band has been allocated in Japan, and an experimental mobile WiMAX system was developed in Azumino City in Japan [6, 7]. A network service and applications have been provided to citizens within the local wireless network area. Base stations (BSs) complying with the mobile WiMAX based on IEEE 802.16e standard [8] were installed in 2009 and 2010 to increase the channel quality and channel capacity of the system. As previous work, the basic throughput performance in a field experiment under a static condition of MU-MIMO transmission with the mobile WiMAX system was reported [9], and the RSSI and throughput performances within 500 m from the BS were measured [10]. In this paper, the transmission performance for downlink MU-MIMO systems based on the experimental mobile WiMAX system is evaluated with a computer simulation and field experiment. A MU-MIMO system based on the experimental mobile WiMAX system is constructed as a transmitter with 6 antennas and receivers with 2 antennas, and the number of streams is 1-2. In computer simulation, a MU-MIMO transmission system can be realized by using the block diagonalization (BD) algorithm. Transmission performances are evaluated in terms of bit error rate (BER) performance and a channel capacity in a spatially correlated multipath fading environment. Furthermore, in such an environment, we propose a method for evaluating transmission performance for the mobile WiMAX system with MU-MIMO by using the computer simulation. In the field experiment, received power and throughput performances for the downlink experimental mobile WiMAX system with MU-MIMO are measured. We show the transmission performance with the proposed evaluation method for the mobile WiMAX system with MU-MIMO under the spatially correlated multipath fading environment.

This paper consists of the following sections. In Section 2, we describe a system model and procedures for MU-MIMO transmission with the BD algorithm. In Section 3, we describe the results of the computer simulation and details of the proposed evaluation method, and in Section 4, we describe and analyze the results of the field experiment. In Section 5, we discuss the result of computational and field experimental results on interference. Finally, Section 6 concludes this paper.

## 2. Downlink MU-MIMO System Model

**Figure 1:**
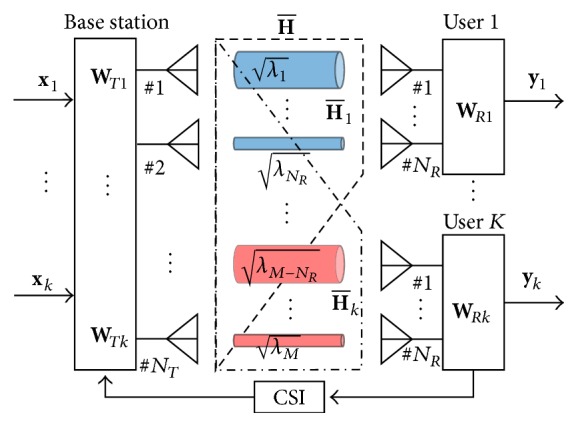
System model for downlink MU-MIMO system.

A downlink mobile WiMAX system model based on the IEEE 802.16e standard is constructed in this paper. The model with MU-MIMO technique is shown in Figure 1. *N*_*T*_ denotes the number of transmitting antennas at the base station, and *N*_*R*_ denotes the number of receiving antennas at a user. The maximum number of transmitting streams is 3; therefore, the number of users is 1–3 in the computer simulation described in Section 3. The number of transmitting and receiving antennas is determined with the experimental mobile WiMAX system in the field experiment described in Section 4.

In Figure 1, **x**_*k*_ and **y**_*k*_, respectively, denote the input and output signals for *k*th user, where *k* = 1,2,…, *K*. In the MU-MIMO transmission for the *k*th user, the input signal is multiplied by a weight matrix at the transmitter for the *k*th user **W**_*Tk*_; then, it is transmitted to the user. The received signal for the *k*th user **r**_*k*_ is also multiplied by a weight matrix at the receiver for the *k*th user **W**_*Rk*_; then, output signal is obtained.

In this paper, we assumed that the MU-MIMO system is based on the OFDM system and that the subcarriers are influenced by flat fading through channels. When an input signal **x**_*k*_ for the *k*th user is transmitted in this situation, the received signal for *k*th user is given by

(1) $$\begin{array}{c} r_{k}=H_{k}\overset{K}{\underset{i=1}{\sum }}W_{Ti}x_{i}=H_{k}W_{Tk}x_{k}+H_{k}\overset{K}{\underset{i=1,i\neq k}{\sum }}W_{Ti}x_{i}. \end{array}$$

Here, noise components are ignored. In this equation, *H*_*k*_ denotes the MIMO channel state matrix for the *k*th user, and *K* denotes the maximum number of users. Here, we assumed that the MIMO channel state information is perfectly received by the transmitter from the receivers.

The output signal for *k*th user **y**_*k*_ is given by

(2) $$\begin{array}{c} y_{k}=W_{Rk}r_{k}=W_{Rk}H_{k}W_{Tk}x_{k}+W_{Rk}H_{k}\overset{K}{\underset{i=1,i\neq k}{\sum }}W_{Ti}x_{i}. \end{array}$$

In (2), the component **W**_*Rk*_ **H**_*k*_ **W**_*Tk*_ **x**_*k*_ denotes the desired signal for the *k*th user, and the other components **W**_*Rk*_ **H**_*k*_∑_*i*=1,*i*≠*k*_^*K*^ **W**_*Ti*_ **x**_*i*_ are the interference signals between the *k*th user and the other *K* − 1 users. Therefore, it is important for MU-MIMO transmission to eliminate the interference signals for each user.

In the conventional approaches for downlink MU-MIMO systems, dirty paper coding (DPC) [11] and BD algorithms [12] are well-known for multiuser detection. However, the DPC algorithm requires extremely high calculation cost. Therefore, to realize MU-MIMO transmission, we used the BD algorithm in this paper. In the MU-MIMO transmission system based on the BD algorithm, which includes linear pre- and postprocessing, the weight matrix at the transmitter must satisfy

(3) $$\begin{array}{c} H_{i}W_{Tj}=0\;\forall i,j\;\left(1\leq i,j\leq K\right). \end{array}$$

Here, we assumed a new MIMO channel state matrix except for the *k*th user as follows:

(4) $$\begin{array}{c} {\tilde{H}}_{k}=\left[H_{1}^{T}\cdots H_{k-1}^{T}H_{k+1}^{T}\cdots H_{K}^{T}\right], \end{array}$$

where the operator [·]^*T*^ denotes a matrix transpose. The SVD of the matrix ${\tilde{H}}_{k}$ is defined by

(5) $$\begin{array}{c} {\tilde{H}}_{k}={\tilde{U}}_{k}{\tilde{\Sigma }}_{k}{\left[{\tilde{V}}_{k}^{s}{\tilde{V}}_{k}^{n}\right]}^{H}, \end{array}$$

where the operator [·]^*H*^ denotes the Hermitian matrix transpose. In this equation, term ${\tilde{V}}_{k}^{s}$ denotes the single space of all users except for the *k*th user, and term ${\tilde{V}}_{k}^{n}$ denotes the null space that does not interfere without the other *K* − 1 users.

Because ${\tilde{H}}_{k}{\tilde{V}}_{k}^{n}=0$, we can satisfy the condition in (3) by using the weight matrix ${\tilde{V}}_{k}^{n}$.

After processing the block diagonalization, the MU-MIMO system can be achieved as *K* parallel SU-MIMO transmissions for each user.

In accordance with the SVD-SU-MIMO transmission [13–15] for the *k*th user, the SVD of the SVD-SU-MIMO channel state matrix ${\overset{-}{H}}_{k}=H_{k}{\tilde{V}}_{k}^{n}$ is defined as

(6) $$\begin{array}{c} {\overset{-}{H}}_{k}={\overset{-}{U}}_{k}{\overset{-}{\Sigma }}_{k}{\left[{\overset{-}{V}}_{k}^{s}{\overset{-}{V}}_{k}^{n}\right]}^{H}, \end{array}$$

where ${\overset{-}{U}}_{k}$ and ${\overset{-}{V}}_{k}^{s}$ are the left and right singular vectors, respectively, and ${\overset{-}{\Sigma }}_{k}$ is the diagonal matrix whose elements $\sqrt{\lambda_{i}}$ are the square roots of the null space eigenvalues. Thus, the weight matrix at the transmitter for SVD transmission is given as $W_{Tk}={\tilde{V}}_{k}^{n}{\overset{-}{V}}_{k}^{s}$. By using the weight matrix at the receiver $W_{Rk}={\overset{-}{U}}_{k}^{H}$, the output signal for the *k*th user in (2) is rewritten as

(7) $$\begin{array}{c} y_{k}={\overset{-}{\Sigma }}_{k}x_{k}. \end{array}$$

In this equation, for example, the output signal for user 1 is represented as

(8) $$\begin{array}{c} y_{1}={\overset{-}{\Sigma }}_{1}x_{1}=\left[\begin{array}{ccc} \sqrt{\lambda_{1}} & \cdots & 0\\ \vdots & \ddots & \vdots \\ 0 & \cdots & \sqrt{\lambda_{N_{R}}} \end{array}\right]x_{1}. \end{array}$$

The MU-MIMO transmission without any interference between the *k*th user and the other users can be realized as parallel SVD-SU-MIMO transmission by using the above weight matrices at the transmitter and the receivers, and the quality of each parallel wireless link is different depending on the diagonal elements $\sqrt{\lambda_{i}}$ [3, 13–15].

Summarizing the above procedures, a transmitting weight matrix $W_{Tk}={\tilde{V}}_{k}^{n}{\overset{-}{V}}_{k}^{s}$ is calculated by (5) and (6) by using the SVD operation with CSI. The input signal multiplied by the transmitting weight matrix is transmitted to the users. For each user, the receiving weight matrix $W_{Rk}={\overset{-}{U}}_{k}^{H}$ is calculated by (6) by using the SVD operation. The signal received by each user is multiplied by the receiving weight, and the output signal is obtained for each user.

Note that the above MU-MIMO transmission with BD algorithm can transmit signals only to the desired user without any interference between users under independently identically distributed (i.i.d.) channel conditions. In spatially correlated multipath fading environments, there are some interference values between received signals for each user [16].

## 3. Computer Simulation

### 3.1. Simulated Condition

To evaluate the system performance, we carried out a computer simulation by using the downlink MU-MIMO system shown in Figure 1.  Table 1 lists the parameters for the simulations.

The parameters were determined in accordance with the experimental mobile WiMAX system based on the IEEE 802.16e standard. The downlink access scheme was OFDMA-TDD, and OFDM-QPSK, 16QAM, and 64QAM were used as modulation schemes. The number of transmitting antennas was 6, and the number of receiving antennas at one user was 2. The maximum number of streams was 3. In the MU-MIMO model, the frequency was 2587 MHz, and the bandwidth was 10 MHz. The input streams were modulated as QPSK, 16QAM, and 64QAM modulation scheme, and the number of streams was the same as the number of users. The number of FFT points was 1024. A 12-ray Rayleigh fading model whose maximum delay time is 3.3 *μ*s was used as the channel model, and the Doppler frequency was set to 5 Hz in consideration of transmission at walking speed.

### 3.2. BER Analysis

**Figure 2:**
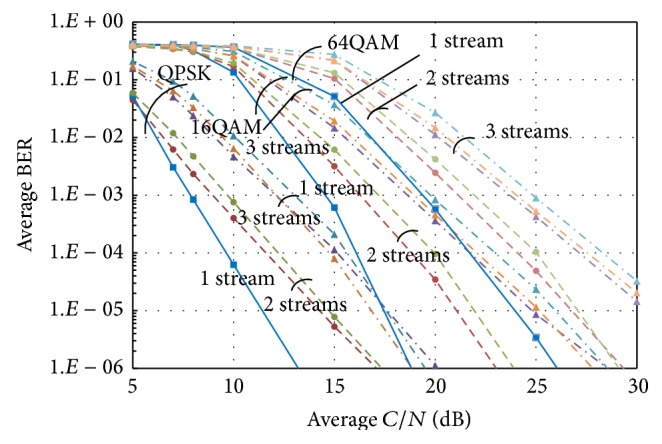
BER performance in accordance with modulation schemes and number of streams.

Figure 2 shows the average *C*/*N* versus the average BER in accordance with the number of streams and the modulation schemes under the multipath fading environment.

In the graph, the average BER performances are plotted for 1 user with 1 stream, for 2 users with 2 streams, and for 3 users with 3 streams in accordance with the modulation schemes. In the case of 1 stream, for example, a system having a BER of 10^−6^ required an average *C*/*N* of 13.2 dB in QPSK. However, the system having the same BER of 10^−6^ required an average *C*/*N* of 16.8 dB when the number of streams was 2. The difference of these required *C*/*N*s is 3.6 dB, and this indicates the interference signal value between signals for the other users. In the case of 3 streams, the system having the same BER of 10^−6^ required an average *C*/*N* of 21.6 dB. The interference values corresponded to *C*/*I* of 15.7 dB and 13.9 dB when the numbers of streams were 2 and 3, respectively.

The same as the result with QPSK modulation, systems having a BER of 10^−6^ required an average *C*/*N* of 20.8, 23.8, and 29.1 dB when the numbers of streams were 1, 2, and 3, respectively, in 16QAM. The interference values corresponded to *C*/*I* of 23.8 dB and 21.5 dB when the numbers of streams were 2 and 3, respectively. Furthermore, systems having a BER of 10^−6^ required an average *C*/*N* of 26.2, 30.0, and 34.7 dB when the numbers of streams were 1, 2, and 3, respectively, in 64QAM. The interference values corresponded to *C*/*I* of 28.5 dB and 26.6 dB when the numbers of streams were 2 and 3, respectively.

### 3.3. Channel Capacity Analysis

**Figure 3:**
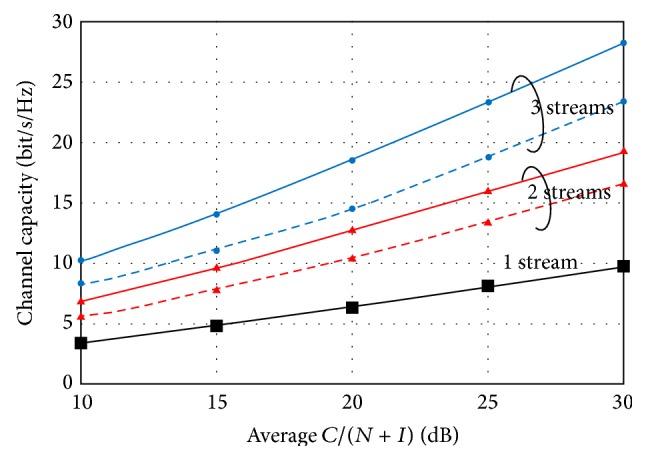
Channel capacity in accordance with spatially correlation and number of streams.

The total channel capacity on the MU-MIMO systems increased as the number of transmitting antennas and streams increased.  Figure 3 shows the average *C*/(*N* + *I*) versus the total channel capacity with the number of streams being 1–3 under the spatially correlated environment. The line and dashed-line in the graph denote the channel capacity under no correlated and spatially correlated Rayleigh fading environments, respectively.

In such an i.i.d. Rayleigh fading condition, the channel capacity based on the BD algorithm is calculated as

(9) $$\begin{array}{c} C_{\text{BD}}=\max\log_{2}I+\frac{{\overset{-}{\Sigma }}^{2}\Lambda }{\delta_{n}^{2}}, \end{array}$$

where

(10) $$\begin{array}{c} \overset{-}{\Sigma }=\left[\begin{array}{ccc} {\overset{-}{\Sigma }}_{1} & \cdots & 0\\ \vdots & \ddots & \vdots \\ 0 & \cdots & {\overset{-}{\Sigma }}_{K} \end{array}\right]. \end{array}$$

The details for calculating *C*_BD_ are described in [17].

Since spatial correlation has played an important role in evaluating the SU-MIMO system, its effect can be applied to the MU-MIMO system. In spatially correlated Rayleigh fading environments, the channel capacity is lower than that in i.i.d. environments [17, 18]. To evaluate the transmission performance for the MU-MIMO system in a spatially correlated environment, we propose an evaluation method that uses computer simulation. Here, we calculated the transmission speed for the downlink mobile WiMAX system in order to evaluate the throughput performance in the field experiment described in Section 4. The downlink bandwidth is 10 MHz and the number of downlink subcarriers for data transmission is 720, so the downlink bandwidth for data transmission is 720/1024 × 10 MHz = 7.0 MHz. The maximum transmission speed for only 1 user in downlink is calculated as

(11) Transmission  Speed=6bit×56×23×7205[ms]=16.17Mbps,

where the primary modulation scheme is 64QAM, the convolutional coding rate is 5/6, and the number of OFDM symbols for data transmission per 1 OFDMA/TDD frame is 23.

Because only 23 OFDM symbols are used for downlink data transmission in 47 OFDM symbols per frame, the channel capacity per frequency with 1 stream can be calculated by

(12) $$\begin{array}{c} W_{1}=\frac{16.17\left[\text{Mbps}\right]/7\left[\text{MHz}\right]}{\left(23/47\right)}=4.72\left[\text{bps}/\text{Hz}\right]. \end{array}$$

Here, the average *C*/(*N* + *I*) value corresponds to about 15 dB as shown in Figure 3, and the channel capacity becomes depleted because the interference value cannot be eliminated completely if the number of streams is larger than 2. Under this spatially correlated condition, the channel capacity per frequency with 2 streams *W*_2_ and 3 streams *W*_3_ was about 8.0 bps/Hz and 12.1 bps/Hz, respectively. Thus, it was converted to the transmission speed of 27.4 Mbps for 2 streams and 41.4 Mbps for 3 streams. In the field experiment, we used the simulated results of 16.17 Mbps for 1 stream and 27.4 Mbps for 2 streams for the evaluation because the maximum number of streams for the experimental mobile WiMAX system was 2.

## 4. Field Experiment

### 4.1. Overview of Experimental Mobile WiMAX System

**Figure 4:**
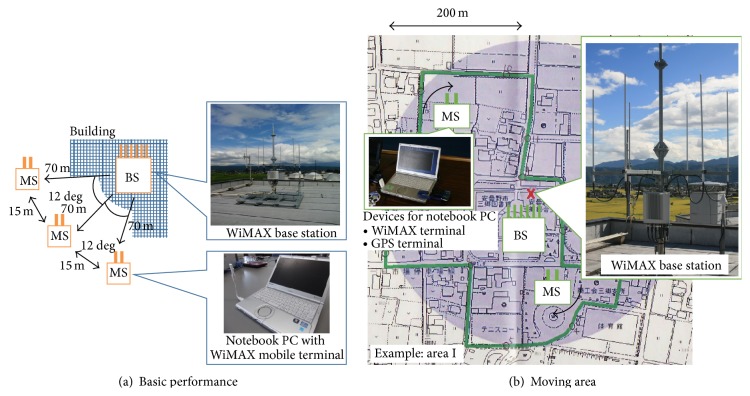
Setup for field experiment.

Figure 4 shows an overview of the experimental mobile WiMAX system constructed in Azumino City in Japan. In the network area of the system, we measured the received power and the throughput performances for MU-MIMO transmission within 200−500 m centering on the BS.

A WiMAX BS complying with IEEE 802.16e was used, and the parameters are listed in Table 1. The frequency was 2587 MHz, which is an open frequency band for local communities, and the bandwidth was 10 MHz and called the “local band.” The frame structure was OFDMA/TDD, and its interval was 5 ms, which was decided by the system profile of the mobile WiMAX. The ground height of the BS was about 17 m. Here, CSI feedback was transmitted from the MSs to the BS by using the codebook algorithm [19].

The number of transmitting antennas at the BS is 6, and the number of receiving antennas at a MS was 2. The number of users was 1-2 because the maximum number of streams in the WiMAX system with Matrix A/B mode was 2. In Matrix A mode, the maximum number of streams is 1, and the system can obtain the transmitting diversity gain to improve the channel quality. In Matrix A/B mode, the maximum number of streams is 2. The number of streams in Matrix A/B mode is changed into 1 or 2 dynamically, and the system can increase the total channel capacity by using the MU-MIMO technique when the number of streams is 2.

The received power and the throughput of downlink were measured by using a notebook PC. Users each had a notebook PC with a WiMAX terminal device and GPS terminal devices, and the samples of the received power, throughput, and position were measured. The throughput performance in the UDP layer was measured by using the same notebook PC connected to the WiMAX BS directly. The interval getting the samples was 1 second.

In the field experiments, at first, throughput performance is measured under the static and LOS conditions in order to compare the basic performance with MU-MIMO transmission with Matrix A and Matrix A/B modes. An overview of the basic performance is shown in Figure 4(a).

Then, as shown in Figure 4(b), the received power and throughput performance were measured by walking in “Area I” and “Area II.” These areas were within 200 and 500 m from the experimental mobile WiMAX BS, respectively. In them, there were many positions under line-of-sight (LOS) and non-LOS (NLOS) conditions because of buildings and trees. When the number of streams was 1, only 1 user was walked, and samples were measured. When the number of streams was 2, 2 users were walked, and samples were measured at the same time.

### 4.2. Extension for MU-MIMO from SU-MIMO

To evaluate the basic performance of downlink MU-MIMO transmission based on the experimental mobile WiMAX system, we measured throughput performance with Matrix A and Matrix A/B modes under static and LOS conditions.

**Figure 5:**
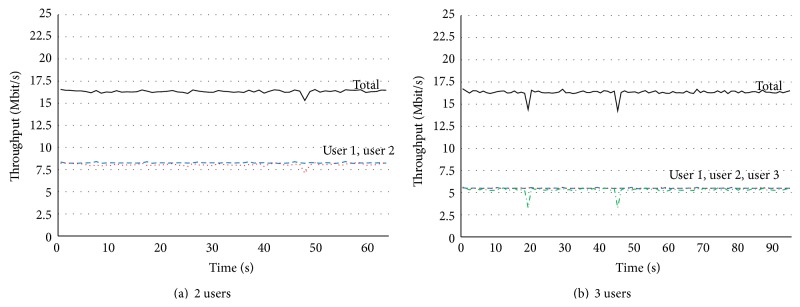
Basic throughput performance with Matrix A mode.

Figure 5 shows the measured throughput performance with Matrix A mode, and the numbers of users were 2 in (a) and 3 in (b). From these graphs, the maximum total throughput in (a) and (b) was the same value. Because Matrix A mode can only improve the channel quality, the channel capacity cannot be increased. As shown in (a), the measured throughputs for users 1 and 2 were about 8 Mbps, and the measured total throughput was about 16 Mbps. As shown in (b), the measured throughputs for each user were about 5.3 Mbps. In Matrix A mode, each user can be provided almost the same throughput performance.

**Figure 6:**
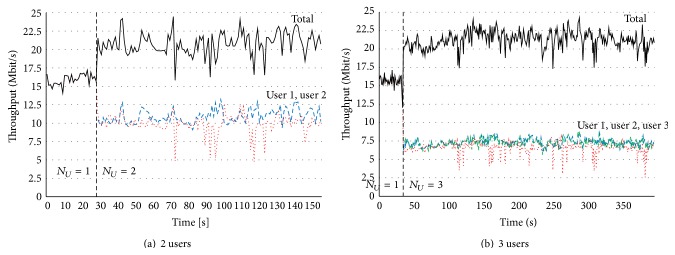
Basic throughput performance with Matrix A/B mode.

Figure 6 shows the measured throughput performance with Matrix A/B mode, and the number of users were 2 in (a) and 3 in (b). Comparing the maximum throughput with Matrix A mode and Matrix A/B mode, the maximum throughput with Matrix A/B mode was higher than that with Matrix A mode. Because Matrix A/B mode can increase the total channel capacity, the channel capacity in accordance with the number of streams can be obtained. In these graphs, first, the throughput was measured only with 1 user within 30 seconds. After a lapse of 30 seconds, throughputs with 2 and 3 users were measured. The results in Figure 6 also show that the maximum throughput with Matrix A/B mode, whose number of streams was 2, was about 23 Mbps, and the throughputs for each user were almost the same values.

### 4.3. Received Power Performance

**Figure 7:**
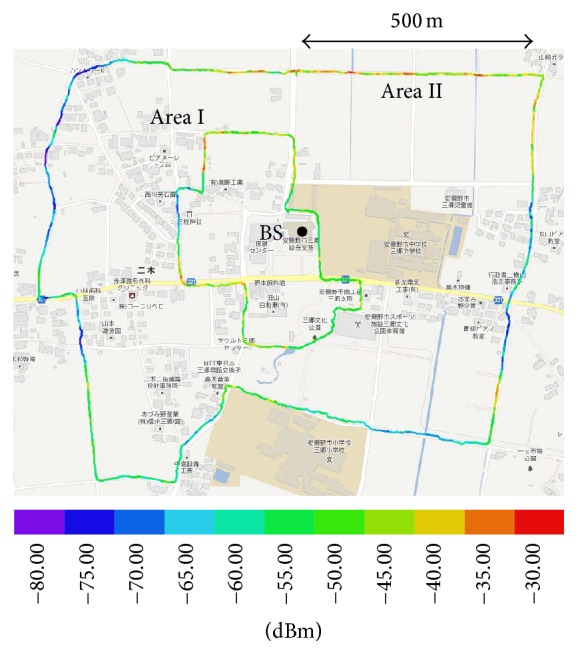
Route map and received power at each point.

**Figure 8:**
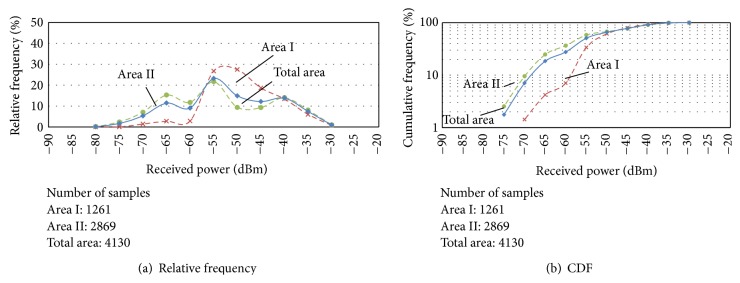
Measured received power distribution.

Figure 7 shows both a route map and the received power at each point on the moving route, and Figure 8 shows the result of the measured received power versus the relative frequency in (a) and the cumulative frequency in (b).

In Area I, the maximum received power was about −30 dBm, the minimum was about −70 dBm, and the mode value was about −50 dBm. In Area II, the maximum received power was about −35 dBm, the minimum was about −75 dBm, and the mode value was about −55 dBm. In total area, the mode value was about −55 dBm. The distance between the WiMAX BS and the positions in Area II was longer than that in Area I, so that the received power in Area II was lower than that in Area I. Compared with the CDF of received power in (b), the received powers were −53, −58, and −57 dBm when the CDF in Area I, Area II, and the total area became 50%, respectively. The received powers were −58, −70, and −68 dBm when the CDF in Area I, Area II, and the total area became 10%, respectively. Moreover, the CDF of received power in Area I was 10 dBm, almost single-digit, decreasing to under −55 dBm, and the CDF of received powers in both Area II and the total area was 10 dBm, almost single-digit, decreasing to under −65 dBm. Therefore, the received and transmitted signals in the total area were under the influence of the fading environment.

### 4.4. Received Throughput Performance

**Figure 9:**
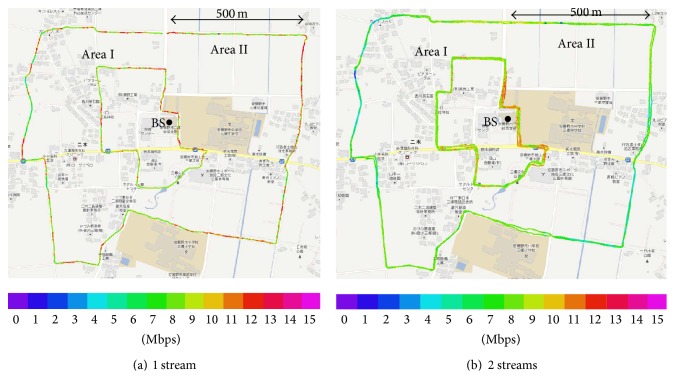
Route map and measured throughput at each point.

**Figure 10:**
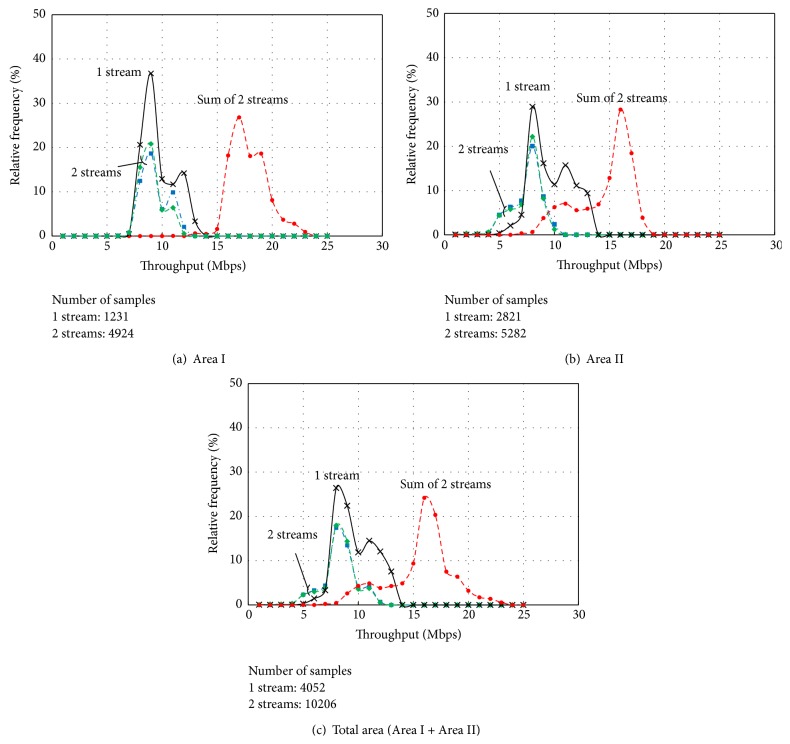
Throughput performance distribution in accordance with areas and number of streams.

Figure 9 shows both a route map and the measured throughput at each point on the moving route.  Figure 10 shows the result of the measured throughput versus the relative frequency under Area I in (a), Area II in (b), and the total area in (c).

In each graph, “1 stream” denotes the measured throughput with only 1 user and 1 stream. “2 streams” denotes the measured throughput with 2 users and 2 streams, and the throughputs of each user are plotted. “Sum of 2 streams” denotes the sum of the throughputs of 2 users at the same time.

#### 4.4.1. Area I

When the number of streams was 1, the maximum throughput was 13 Mbps, the minimum was 8 Mbps, and the mode value of throughput was 9 Mbps, as shown in Figure 10(a). When the number of streams was 2, the maximum was 12 Mbps, and the minimum was 8 Mbps for each user. The throughputs for each user were distributed similarly. We summarized the throughputs for each user at the same time, and the maximum and the minimum throughputs were 23 and 15 Mbps, respectively.

#### 4.4.2. Area II

When the number of streams was 1, the maximum throughput was 13 Mbps, the minimum was 5 Mbps, and the mode value of throughput was 8 Mbps, as shown in Figure 10(b). When the number of streams was 2, the maximum throughput was 10 Mbps and the minimum was 4 Mbps for each user. The throughputs for each user were also distributed similarly. The maximum and the minimum throughputs, summarizing the throughputs for each user at the same time, were 18 and 7 Mbps, respectively. Comparing between Areas I and II, the received power in Area II was lower than that in Area I, so that the throughput in Area II was also lower than that in Area I.

#### 4.4.3. Total Area

Finally, Figure 10(c) shows the result in the total area (Area I + Area II). When the number of streams was 1, the maximum throughput was 13 Mbps, the minimum was 5 Mbps, and the mode value of throughput was 9 Mbps. When the number of streams was 2, the throughputs for each user were distributed as the same pattern, and the maximum throughput was 12 Mbps, the minimum was 4 Mbps, and the mode value of throughput was 8 Mbps. The maximum and minimum throughputs, summarizing the throughputs for each user at the same time, were 23 Mbps and 7 Mbps, and the mode value was 16 Mbps.

Note that the downlink maximum throughputs in the physical layer calculated in the above section were 16.2 Mbps and 27.4 Mbps when the numbers of streams were 1 and 2, respectively. The maximum measured throughputs were 13 Mbps and 23 Mbps in the UDP layer; therefore, the throughputs for only 1 and 2 streams were almost the same values as the simulated throughputs in Section 3.3. Furthermore, the MU-MIMO system with 2 streams has twice the throughput performance as compared with that with 1 stream if the system can eliminate interference between signals for the other users perfectly. Although there was some interference between the users, the total throughput performance with 2 streams can be improved by the beamforming in all areas. Therefore, it was confirmed that the MU-MIMO transmission system based on the mobile WiMAX was successfully constructed, and increasing and evaluating the total channel capacity on the system were successfully performed.

## 5. Discussion

In the computer simulation, we assumed that the transmitter can receive channel information perfectly with CSI feedback from the receivers. However, in the actual field environment, channel information feedback with codebook algorithm [19] was constructed. Such channel information with the codebook algorithm is not perfectly equal compared with the simulated condition. These errors of such feedback CSI cause the interference values between signals for each user, and the sum of throughput performances for 2 users is not equal to twice the throughput for 1 user. The influence caused by the interference should be analyzed in field experiments. For example, the performance of beamforming gains with the experimental WiMAX BS is described and analyzed in [20]. To analyze the interference between the signals for 2 users, beamforming gains should also be measured in the future.

Although there are few interference values for MU-MIMO transmission with the experimental mobile WiMAX system, the improvement of the total throughput shows that MU-MIMO transmission was realized with the experimental mobile WiMAX system. Furthermore, under spatially correlated multipath fading environments the throughput performance can be preindicated by using the simulated results.

## 6. Conclusion

We evaluated the transmission performance for a downlink MU-MIMO system by computer simulation and in a field experiment. For the field experiment, an experimental mobile WiMAX system was constructed based on the IEEE 802.16e standard, and the BS had 6 transmitting antennas. The numbers of streams in the computer simulation and field experiment were 1–3 and 1-2, respectively, and the MSs had 2 receiving antennas for each user. A MU-MIMO system model in the computer simulation was constructed in accordance with the experimental mobile WiMAX system, and MU-MIMO transmission based on the BD algorithm was performed. In the computer simulation, BER performance and channel capacity were analyzed under spatially uncorrelated and correlated multipath fading environments. The results show that the interference values between signals for the other users influenced the BER performance and throughput performance. Furthermore, we proposed a method for evaluating transmission performance for the mobile WiMAX system with MU-MIMO under a spatially correlated multipath fading environment. In the field experiment, the received power and downlink throughput performance were measured by walking around areas. The results show that the maximum downlink throughput with 1 stream was about 13 Mbps and the maximum total throughput with 2 streams was about 23 Mbps. Therefore, it was confirmed that MU-MIMO transmission based on mobile WiMAX successfully confirmed increased the total channel capacity of the system. Moreover, the experimental throughput performance could be evaluated correctly by using the proposed evaluation method.

## Figures and Tables

**Table 1:** Parameters for experimental mobile WiMAX system.

Parameters	Value
Frequency	2587 [MHz]
Bandwidth	10 [MHz]
Modulation scheme	QPSK/16QAM/64QAM
Convolutional code	1/2, 3/4, 5/6
Number of FFT points	1024
Effective OFDM symbol length	91.4 [*μ*s]
CP length	11.4 [*μ*s]
OFDM symbol length	102.9 [*μ*s]
Frame structure	OFDMA-TDD
Frame interval	5 [ms]
Number of OFDM symbols per frame	47
OFDM symbol ratio (DL : UL)	29 : 18
OFDM symbol ratio for data (DL : UL)	23 : 15
Number of transmitting antennas	6
Number of receiving antennas	2
Maximum number of streams	1 (Matrix A mode)
	2 (Matrix A/B mode)
Channel model	12-ray Rayleigh fading model
	(Maximum delay is 3.3 [*μ*s])
Doppler frequency	5 [Hz]
Mode	Matrix A, Matrix A/B

Parameters for PUSC	DL/UL

Number of data subcarriers	720/560
Number of subchannels	30/35
Number of OFDM symbols per slot	2/3
